# A Method for Identification of Biotype-Specific Salivary Effector Candidates of Aphid

**DOI:** 10.3390/insects14090760

**Published:** 2023-09-13

**Authors:** Duoqi Wang, Qinglan Yang, Xiaoyue Hu, Biao Liu, Yongmo Wang

**Affiliations:** 1 Hubei Insect Resources Utilization and Sustainable Pest Management Key Laboratory, College of Plant Science & Technology, Huazhong Agricultural University, Wuhan 430070, China; 13292899023@163.com (D.W.); y1751118447@163.com (Q.Y.); huxiaoyue971202@163.com (X.H.); 2 Nanjing Institute of Environmental Science, Ministry of Ecology and Environment of the People’s Republic of China, Nanjing 210042, China; liubiao@nies.org

**Keywords:** aphid–plant interaction, effector, host specialization, proteome, salivary protein, transcriptome

## Abstract

**Simple Summary:**

Aphids are generally dietary specialists, colonizing a specific plant or a group of closely related plants, but a few species are generalists, colonizing hundreds of hosts across multiple plant families. In these generalist aphids, host-specialized lineages or host-specialized biotypes are often observed in nature. This is the case for the cotton-melon aphid, *Aphis gossypii* Glover. When introduced to alternative hosts, the host-specialized biotypes show poor fitness and may even die within a few days. The underlying mechanisms of aphid host specialization remain unknown until now. We hypothesized that host-specialized biotypes express biotype-specific salivary effectors or elicitors that determine the compatibility of aphid-plant interactions. In this research, we described three strategies to identify biotype-specific effectors in two host-specialized biotypes of *A. gossypii*, a biotype specialized in Malvaceae and another in Cucurbitaceae. The strategy of combining transcriptome and proteome has the highest efficiency, obtaining less than one dozen effector candidates, and we strongly recommend this strategy to identify biotype-specific effectors in aphids and other sap-sucking insects.

**Abstract:**

Polyphagous aphids often consist of host-specialized biotypes that perform poorly in non-native hosts. The underlying mechanisms remain unknown. Host-specialized biotypes may express biotype-specific salivary effectors or elicitors that determine aphid hosts. Here, we tried three strategies to identify possible effectors in Malvaceae- (MA) and Cucurbitaceae-specialized (CU) biotypes of the cotton-melon aphid *Aphis gossypii* Glover. The whole-aphid RNA-seq identified 765 differentially expressed genes (DEGs), and 139 of them were possible effectors; aphid-head RNA-seq identified 523 DEGs were identified, and 98 of them were possible effectors. The homologous genes of published aphid effectors were not differentially expressed between CU and MA. Next, quantitative proteomic analyses of saliva identified 177 possible proteins, and 44 of them were different proteins. However, none of the genes of the 44 proteins were differentially expressed, reflecting the discrepancy between transcriptome and proteome data. Finally, we searched for DEGs of the 177 salivary proteins in the aphid-head transcriptomes, and the salivary proteins with expression differences were regarded as effector candidates. Through this strategy, 11 effector candidates were identified, and their expression differences were all confirmed by RT-qPCR. The combinatorial analysis has great potential to identify biotype-specific effector candidates in aphids and other sap-sucking insects.

## 1. Introduction

Aphids are a special group of insect herbivores that pump liquid nutrients through their special piercing and sucking mouthparts from plants [1]. As agricultural and horticultural pests, aphids inflict damage directly by removing phloem sap and indirectly by vectoring plant viruses and causing sooty mold [1]. Among the 4000+ identified aphid species, the vast majority are specialists that feed on only a small number of closely related plant species, but a few are extreme generalists that infest hundreds of hosts across multiple plant families [2]. However, among generalist aphids, such as the cotton-melon aphid *Aphis gossypii* Glover, host-specialized biotypes or lineages were often observed in nature [3,4,5]. When being introduced to non-native hosts, especially of different families, host-specialized biotypes performed poorly and even died within a few days [6]. To date, the mechanisms of aphid host specialization still remain to be elucidated. 

During feeding, aphids inject saliva that is comprised of a variety of proteins and other small molecules into plants [7,8]. There is increasing evidence that aphid saliva plays an important role in the establishment and maintenance of a compatible aphid–plant interaction [9,10]. When being attacked, plants’ immune surveillances recognize the conserved Herbivore-Associated Molecular Patterns (HAMPs) (so-called insect-associated elicitors) in aphid saliva to start HAMP-triggered immunity (HTI), such as the production of reactive oxygen species (ROS), sieve tube occlusion and biosynthesis of phytoalexins [11,12,13]. HTIs were generally enough to prevent infestation of most aphid species, resulting in incompatible aphid–plant interactions [14,15,16]. However, if an aphid expresses salivary proteins that can suppress HTIs, a compatible aphid–plant interaction will be achieved. 

The salivary protein C002 was the first identified aphid effector that facilitated the feeding and reproduction of *Acyrthosiphon pisum* [17]. Since then, a number of aphid salivary effector proteins were identified, such as Armet [18], Mp1 [19], Mp2, Mp10 [20], Me47 [21], Me10, Me 23 [21]. Subsequent studies found that aphid effectors took effects in a host-specific manner [22,23]. For instance, the reproduction of *M. persicae* improved on genetically modified *Arabidopsis* expressing the *M. persicae* effectors C002, PIntO1 (Mp1), and PIntO2 (Mp2), whereas *M. persicae* reproduction did not increase on *Arabidopsis* expressing the *A. pisum* orthologs of the proteins [24]. In addition, a study reported that aphid effectors were specific not only to aphid species but also to plant species. The fecundity of the potato aphid *Macrosiphon euphorbiae* was enhanced on *Nicotiana benthamiana* expressing the *M. euphorbiae* factor Me23 and not on tomato expressing Me23 [21]. Those findings implied that the host-specialization of an aphid might result from the differential expression of effectors among different biotypes. A recent study identified at least four candidate effectors that were expressed in the alfalfa biotype but not in the pea biotype of *A. pisum* [25]. 

The cotton-melon aphid *A. gossypii* is an extremely polyphagous aphid pest, infesting more than 900 plants across 116 plant families [1]. There were several host-specialized biotypes in this aphid, among which the Cucurbitaceae biotype (CU) and Malvaceae biotype (MA) are specialized in cucurbits and Malvaceae, respectively, were most studied. Parthenogenetic *A. gossypii* from cotton (MA biotype) did not survive in cucumber and pumpkin, and those from cucumber (CU biotype) did not survive or perform poorly in cotton [26,27,28,29]. Different from the pea aphid *A. pisum,* whose biotypes specialize in different legumes, *A. gossypii* biotypes specialize in plants across different families. In addition, a high-quality genome assembly of *A. gossypii* has recently been published [30,31]. Therefore, *A. gossypii* is an ideal model to test the hypothesis of the biotype-specific effectors.

Genomic, transcriptomic, and proteomic approaches or their combinations can be used to identify putative effectors of aphids [32]. Effector candidates were highly expressed in salivary glands compared to other organs [17], so identifying differentially expressed genes (DEGs) by comparative transcriptome analysis between salivary glands and other organs was also used [33,34]. The characteristics of secreted proteins, such as having predicted signal peptides and having no predicted transmembrane regions [32,35], can be used to enrich effector candidates. Significant advances in high-resolution mass spectrometry (MS) provide new opportunities to identify proteins directly from trace saliva collected from an artificial diet [35,36]. Proteomic approaches were highly efficient and usually generated a small catalog of effector candidates. In the present study, we tried three strategies to identify biotype-specific effector candidates using MA and CU biotypes of *A. gossypii* as insect materials. We established an efficient and low-cost strategy that can be used to identify biotype-specific effectors in aphids and other sap-sucking insects.

## 2. Materials and Methods

### 2.1. Insect Materials

The Cucurbitaceae-specialized biotype (CU) and Malvaceae-specialized biotype (MA) of *A. gossypii* were collected from cucumber (*Cucumis sativus* L.) and cotton (*Gossypium hirsutum* L.) plants, respectively. Six clonal lines were collected, four of which (CU_1 and CU_2; MA_1 and MA_2) were collected from Baoding (38°53′ N; 115°28′ E), China, in 2015, and two of which (CU_3 and MA_3) from Wuhan (30°48′ N; 114°37′ E), China, in 2019. Sampling sites were more than 1 km apart to attempt to avoid duplicate clones. The aphids were maintained in their native hosts in nylon net cages (45 cm× 45 cm) under 20 ± 2 °C and 16L:8D photoperiod. To avoid overcrowding, aphids were transferred to insect-free plants every two weeks. Insect-free cotton (Huazaimian No. 3) and cucumber (Haiyang) plants were cultivated with nursery soil in square pots (10 cm × 10 cm) in a separate growth room under 25 ± 2 °C and 16L:8D photoperiod. 

Host-specialization of the clonal lines was tested using reciprocal host transfer experiments. To obtain aphids of the same age, five wingless adults were introduced to their native host plants at the two-leaf stage and were removed 24 h later with less than 20 nymphs left in a plant. Five-day-old nymphs of each biotype were introduced to insect-free cucumber and cotton plants at a density of 10 aphids per plant. The plants with aphids were placed in a transparent nylon net cage (0.16 mm mesh size) and maintained in a growth room under 20 ± 2 °C and 16L:8D photoperiod. The number of aphids in each plant was counted every two days. 

### 2.2. Identifying Effector Candidates by RNA-Seq

Two sets of RNA-seq, whole-aphid and aphid-head, were carried out separately. Six-day-old aphids were frozen wholly in liquid nitrogen and were stored at −80 °C for the whole-aphid RNA-seq. To avoid the heavy task of salivary gland dissection, we chose to isolate aphid heads for the aphid-head RNA-seq. Heads of six-day-old aphids were cut off using a knife blade and were soaked in 100 μL RNA later (QIAGEN, Hilden, Germany) immediately. The head samples were then frozen in liquid nitrogen and stored at −80 °C. On average, 5 aphids or 200 aphid heads were pooled for a replicate. Each clonal line was regarded as a biological replicate.

RNA sequencing was conducted by Shanghai Meiji Biomedical Technology Co., Ltd. (Shanghai, China). A TruSeq™ RNA Sample Preparation kit from Illumina (San Diego, CA, USA) was used to prepare RNA-seq libraries. A SuperScript Double-stranded cDNA Synthesis kit (Invitrogen, Carlsbad, CA, USA) was used to synthesize double-stranded cDNA using random hexamer primers (Illumina). According to Illumina’s library construction protocol, the synthesized cDNA was subjected to end-repair, phosphorylation, and ‘A’ base addition. Libraries were size selected for cDNA target fragments of 200–300 bp in 2% low-range ultra-agarose followed by PCR amplification for 15 PCR cycles. After quantification, paired-end RNA-seq libraries were sequenced using the Illumina HiSeq xten/NovaSeq 6000 Sequencer (San Diego, CA, USA). Raw sequencing data were deposited in the NCBI Sequence Read Archive with reference number PRJNA899574.

Gene expression was analyzed using the *A. gossypii* Reference Genome Assembly (GCF_004010815.1). The paired-end libraries were mapped on the reference genome using STAR v2.5.2 [37] with the following parameters: outFilterMultimapNmax = 5, outFilterMismatchNmax = 3, alignIntronMin = 10, alignIntronMax = 50,000, alignMatesGapMax = 50,000. Fragment counts per gene were estimated by Subread featureCounts [38] using default parameters. The expression level of each transcript was calculated according to the fragments per kilobase of exon per million mapped reads (FPKM) to identify differentially expressed genes (DEGs) between biotypes. We used Expectation–Maximization (RSEM) (http://deweylab.biostat.wisc.edu/rsem/, accessed on 10 July 2023) to quantify gene transcript abundances. The R package EdgeR (http://www.bioconductor.org/packages/2.12/bioc/html/edgeR.html, accessed on 10 July 2023) was used for differential expression analysis. Differential expression analysis was performed using the DESeq2 with Q value ≤ 0.05, and the level of up/down-regulation was considered to be significant if equal to greater than two-fold. 

DEGs were subsequently predicted for signal peptide using the online SignalP v4.1 (https://services.healthtech.dtu.dk/service.php?SignalP-4.1, accessed on 12 May 2022) [39] and for transmembrane domains using online TMHMM v2.0 (https://services.healthtech.dtu.dk/service.php?TMHMM-2.0, accessed on 12 May 2022) [40]. DEGs with secretion signals and without membrane insertion domains were defined as effector candidates. Based on 15 published aphid effectors (see Table 1), we used the tBLASTn program (https://blast.ncbi.nlm.nih.gov/Blast.cgi, accessed on 21 March 2022) to search homologous genes in *A. gossypii* (Aphis gossypii: 80765). All sequences in the BLAST hits or with high homologies were manually eliminated selectively as different parts of the same gene or allelic variants. In order to determine whether the homologous genes of published aphid effectors were involved in the host specialization of *A. gossypii*, the hit genes were checked for transcriptional differences in the aphid head transcriptomes of CU and MA biotypes.

### 2.3. Identifying Candidate Effectors by Salivary Proteome

The saliva of CU and MA biotypes was collected by the two-layered Parafilm sandwich method [41]. Aphids were reared in cucumber and cotton plants at two- or three-leaf stages at low density to prevent the development of winged aphids. Approximately 1000 wingless aphids of mixed ages were introduced to a glass tube (φ 4.5 cm, height 6 cm) loaded with 5 mL of 10% sucrose solution in a Parafilm sandwich at one end. The tubes with aphids were kept at 20 °C and 16L:8D photoperiod. The sucrose solution was harvested in 24 h. Daily harvested sucrose solution from each clonal line was pooled as a replicate and stored at −80 °C for later use. 

A replicate was approximately 100 mL of sucrose solution containing saliva from about 20,000 aphids. Filter-aided sample preparation (FASP) method was used for the on-filter digestion of proteins. Protein concentrates (300 μg) in an ultrafiltration filtrate tube (30 kDa cut-off) were mixed with 200 μL UA buffer (8 M urea, 150 mM Tris-HCl, pH 8.0) and centrifuged at 14,000× *g* at 4 °C for 30 min. Then, 100 μL of 50 mM iodoacetamide in UA buffer was subsequently added to the filter, and the samples were then incubated for 30 min at room temperature, followed by centrifugation at 14,000× *g* for 30 min. The filters were washed thrice with 100 μL of UA buffer and centrifuged after each wash. The protein suspensions were digested with 40 μL of trypsin buffer (Promega, Madison, WI, USA) at 37 °C for 18 h. The filter unit was transferred to a new tube, added 40 μL dissolution buffer, and centrifuged at 14,000× g at 4 °C for 30 min. The resulting peptides were collected as a filtrate, and the peptide concentration was quantified at OD280.

The samples of peptides were analyzed by the Easy-nLC nanoflow HPLC system connected to an Orbitrap Elite mass spectrometer (Thermo Fisher Scientific, San Jose, CA, USA). A total of 1 μg of each sample was loaded onto the Thermo Scientific EASY column using an autosampler at a flow rate of 200 nL/min. The sequential separation of peptides on the Thermo Scientific EASY trap column and analytical column was accomplished using a segmented 2 h gradient from 5–28% Solvent B (0.1% formic acid in 100% ACN) for 90 min and then by 28–35% Solvent B for 10 min, 35–90% Solvent B for 2 min, and then 90% Solvent B for 18 min. The mass spectrometer was operated in positive ion mode, and MS spectra were acquired over a range of 350–2000 m/z. The MS scan and MS/MS scan resolving powers were set as 60,000 and 15,000, respectively. The maximum ion injection times were set at 50 ms for the survey scan and 105 ms for the MS/MS scans, and the automatic gain control target values for Master scan modes were 4 × 10^5^, and MS/MS was 1 × 10^5^. The dynamic exclusion duration was 30 s. 

Search for the fragmentation spectra against the 18,497 sequences in the CF_004010815.1_ASM401081v1_protein.fasta. Peptides with a minimum length of seven amino acids were considered for identification. MaxQuant software (v1.5.1.3) was used to calculate the label-free quantification (LFQ), a measure of protein abundance. The LFQ value, which was obtained by dividing protein intensities by the number of theoretically observable tryptic peptides between 5 and 30 amino acids, was, on average, highly correlated with protein abundance. The relative quantification of proteins corresponding to peptides was achieved by analyzing mass spectrometry data and comparing the signal intensity of specific peptides between samples. A protein was considered to be differentially abundant when it had a *p*-value < 0.05 or *FC* (fold change) > 2 or <0.5. Subsequently, those differential proteins were validated for gene expression in the aphid head transcriptomes. The salivary proteins whose transcriptional differences were consistent with the differences of LFQ were regarded as biotype-specific effector candidates.

### 2.4. Combined Analysis of Transcriptome and Proteome

The quantitative proteomics analysis may miss out on some biotype-specific effectors because the saliva secreted into an artificial diet may be different from that secreted into real plant tissues in protein compositions [42,43]. To make full use of the proteins identified from saliva, we searched for DEGs of the salivary proteins in the aphid head transcriptomes. The salivary proteins whose transcription was significantly different between the two biotypes were regarded as enriched biotype-specific effector candidates. 

RT-qPCR was used to validate the gene expression levels of those biotype-specific effector candidates identified by the multi-omics analysis. TB Green^®^ Premix Ex Taq™ II (Takara, Dalian, China) was used in RT-qPCR. As for the cDNA template, we used PrimeScript RT reagent Kit with gDNA Eraser (Takara, Dalian, China) to extract total RNA from CU_3 and MA_3 for first-strand cDNA synthesis in five biological replicates (*n* = 5). NCBI was used to design specific RT-qPCR primers. All of the primers used are listed in Supplementary Table S1. *Ef1-α* gene was used as the reference gene, the Ct value was recorded, and the relative expression of genes was calculated through the 2^−ΔΔCt^ method (ΔCT = CT target gene − CT reference gene, ΔΔCT = ΔCT sample A − ΔCT sample B). 

### 2.5. Statistical Analysis

The population growth was compared between native and non-native hosts using the independent *t*-test. The independent *t*-test was also used to compare the normalized 2^−ΔΔCt^ values. The IBM SPSS Statistics package (version 19.0; SPSS Inc., Chicago, IL, USA) was used for the independent *t*-tests. The methods of differential analysis in omics data are described above.

## 3. Results

### 3.1. Both CU and MA Biotype Exhibited High Host-Specialization

**Figure 1:**
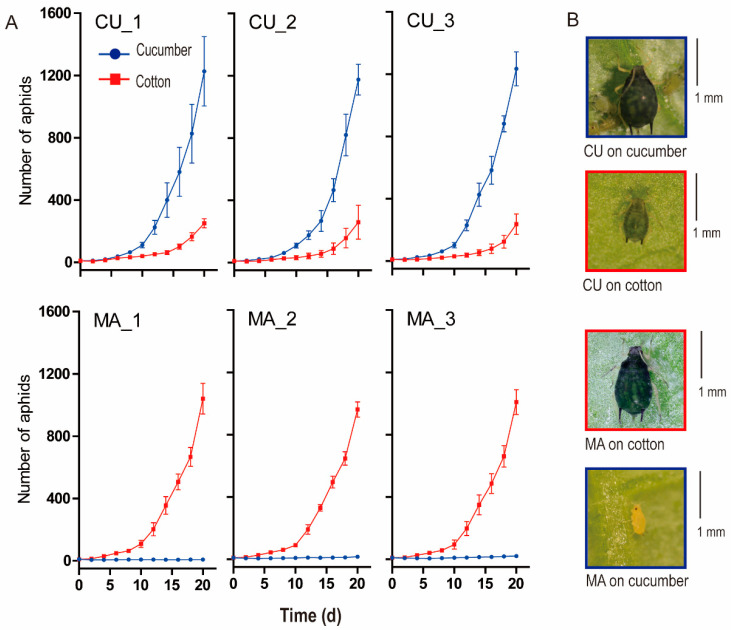
Population growth (**A**) and adult morphology (**B**) of CU and MA biotypes developed in native and non-native hosts. Error bars indicated standard deviation (*n* = 3).

For both CU and MA biotypes, the reproduction in non-native hosts was significantly decreased compared with that in native hosts (*p* < 0.01, *t*-test), and the differences were enlarged with growth time (Figure 1A). On the 20th day, the population of the CU biotype in cotton was 20.48% of that in cucumber, and the population of the MA biotype in cucumber was only 1.49% of that in cotton. The reproduction of the MA biotype in cucumber was significantly lower than that of the CU biotype in cotton since the seventh day of the experiment (*p* < 0.05, *t*-test), indicating the asymmetric host-specialization of the two biotypes. For both biotypes, adults developed in non-native hosts were apparently smaller in body size than that in native hosts, especially for MA biotypes developed in cucumber (Figure 1B). Those results indicated that both biotypes had a high level of specialization to their native hosts, especially the MA biotype to cotton.

### 3.2. Biotype-Specific Candidate Effectors Identified from Transcriptome Analysis

The whole-aphid RNA-seq generated a total of 38.71 Gb clean reads, with at least 5.95 Gb in a single replicate. The percentage of Q30 bases was above 90.93%. Clean data of each sample were mapped against the *A. gossypii* genomic data (GCF_004010815.1), with mapping percentages ranging from 86.41% to 90.28%. A total of 12,904 genes were identified, 11,976 of which were known genes and 928 were new genes. A total of 765 DEGs were detected between the two biotypes, 385 and 380 of which were up-regulated in the CU biotype and MA biotype, respectively. Among the 765 DEGs, 139 had signal peptides, had no transmembrane domains, and were considered candidate effectors (Table S2).

The aphid-head RNA-seq generated 38.71 Gb clean reads. The percentage of Q30 bases was over 90.3%. The mapping percentage ranged from 86.41% to 90.28%. A total of 12,808 genes were identified, 11,929 of which were known genes and 879 were new genes. There were 523 DEGs between the two biotypes, 319 of which were up-regulated in the CU biotype, and 204 were up-regulated in the MA biotype. Among the 523 DEGs, 98 had signal peptides, had no transmembrane domains, and were considered effector candidates (Table S3). Although the aphid-head RNA-seq improved efficiency compared with the whole-aphid RNA-seq, it generated nearly 100 effector candidates.

Through the tBLASTn program, we found homologous proteins of the 15 published aphid effectors in *A. gossypii*, and the percent identified ranged from 32% to 99% (Table 1). None of those homologous proteins were differentially expressed between CU and MA biotypes in either the whole-aphid transcriptome or the aphid-head transcriptome (Table 1). We concluded that those homologous proteins in *A. gossypii* were not the key molecules causing host-specialization of *A. gossypii*.

**Table 1:** Homologous proteins of 15 published aphid effectors in *A. gossypii* and their expression differences between CU and MA biotypes.

Published Effector *	Aphid Species	Homologous Protein ID	Identity (%)	Expression Difference
C002 [17]	*A. pisum*	XP_027848216.1	55	n.s.
Armet [44]	*A. pisum*	XP_027850649.1	95	n.s.
Me10 [21]	*M. euphorbiae*	XP_027842596.1	55	n.s.
Me23 [21]	*M. euphorbiae*	XP_027842036.1	55	n.s.
Me47 [45]	*M. euphorbiae*	XP_027847499.1	54	n.s.
AcDCXR [46]	*A. craccivora*	XP_027848224.1	99	n.s.
Mp1 [22]	*M. persicae*	XP_027842597.1	51	n.s.
Mp10 [47]	*M. persicae*	XP_027847843.1	93	n.s.
Mp55 [47]	*M. persicae*	XP_027849472.1	46	n.s.
Mp56 [47]	*M. persicae*	XP_027850553.1	89	n.s.
Mp57 [47]	*M. persicae*	XP_027846130.1	32	n.s.
Mp58 [47]	*M. persicae*	XP_027842596.1	69	n.s.
ACE1 [48]	*A. pisum*	XP_027838689.1	86	n.s.
ACE2 [48]	*A. pisum*	XP_027838669.1	95	n.s.
ACE3 [48]	*A. pisum*	XP_027850009.1	96	n.s.

* The numbers in parentheses represent references; n.s., not significant.

### 3.3. Biotype-Specific Candidate Effectors Identified by Salivary Proteome

A total of 556 peptides were identified in saliva collected from CU and MA biotypes, and those peptides were mapped to 105 protein groups in the NCBI Non-Redundant Protein Sequence Database of *A. gossypii*. The 105 protein groups represented 177 possible proteins. Among the 105 protein groups, 61 had functional annotations, and 44 were uncharacterized proteins (Table S4). We horizontally compared those salivary proteins with those identified from other aphids, and there were 38 salivary proteins that had been identified in other aphids (Table 2). For example, glucose dehydrogenase was identified in the saliva of nine aphid species, actin in eight other aphid species, carbonic anhydride in seven aphid species, and peroxidase in six aphid species (Table 2). Some uncharacterized proteins identified in *A. gossypii* were also identified in other aphids. For example, the protein LOC114133079, with glucose dehydrogenase activity, was identified in nine aphid species, and the protein LOC114128009, which is homologous to C002 of *A. pisum,* was identified in five other species. Those uncharacterized salivary proteins were repeatedly identified in different aphid species, indicating that they were important in aphid–plant interactions, but their involvement in host specialization was unknown. 

A total of 44 biotype-different protein groups were identified, 26 of which were biotype-unique protein groups, and 18 were biotype-differential protein groups (Table S5). Among the 26 biotype-unique proteins, 23 were unique in the CU biotype, and 3 were unique in the MA biotype (Table S5). Among the 18 biotype-differential proteins, 15 proteins were significantly up-regulated in the CU biotype with FC values ranging from 1.24 to 10.38, and 3 proteins were down-regulated in the CU biotype with FC values ranging from 1.34 to 15.63. Among the 44 protein groups, 27 were characterized proteins, and 24 were uncharacterized proteins. The 44 protein groups were probably the key molecules causing host specialization. However, out of our expectations, none of them had a significant difference in gene expression between CU and MA biotypes. The discrepancy between proteome and transcriptome implied that the saliva collected in an artificial diet might distort the protein compositions of saliva injected into real host plants.

### 3.4. Biotype-Specific Effector Candidates Identified by Combined Analysis

**Figure 2:**
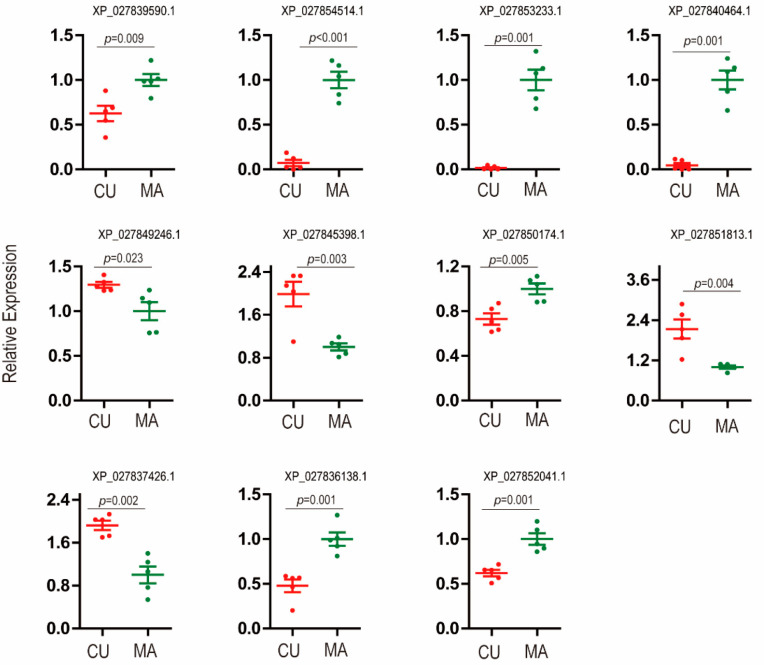
Relative gene expression of 11 biotype-specific effector candidates that were identified by combined analysis of the proteomic and transcriptomic data. Red dots represent the CU biotype, and green dots represent the MA biotype. The vertical bar indicated standard error, and the *p*-value was the significance of the *t*-test (*n* = 5).

Although the quantitative proteome failed to detect biotype-specific effector candidates as expected, the salivary proteins identified by mass spectrometry were deep mined. We searched for DEGs in the aphid-head transcriptomes of the 177 possible salivary proteins in the 105 protein groups. If the corresponding genes of salivary proteins were differentially expressed between CU and MA biotypes, the proteins were regarded as biotype-specific candidate effectors. Through this strategy, we obtained 11 biotype-specific candidate effectors. Among them, seven were down-regulated in the CU biotype, and four were up-regulated in the CU biotype (Table 3). The 11 proteins included actin, three heat shock proteins, a histone protein, an apoptosis-stimulating protein, an E3 ubiquitin-protein ligase, and four proteins with unknown functions. We conducted a phylogenetic analysis using one of the effector candidates, XP027837426.1. The BLASTP in *A. gossypii* detected nine hits, and three of them were effector candidates identified in this study (See Figure S1A); the first 50 hits of BLASTP with no species restriction were all from aphids, and the percent identity ranged from 30.86% to 99.74% (See Figure S1B). The phylogenetic analysis indicated XP027837426.1 is conserved in aphids but variable between aphid species. Although the 11 proteins were identified from aphid saliva, 7 of them had no signal peptide (Table 3). We tested the gene expressions of the 11 proteins by RT-qPCR, and the expression differences and direction of changes were all in line with transcriptomic data (Figure 2).

## 4. Discussion

**Figure 3:**
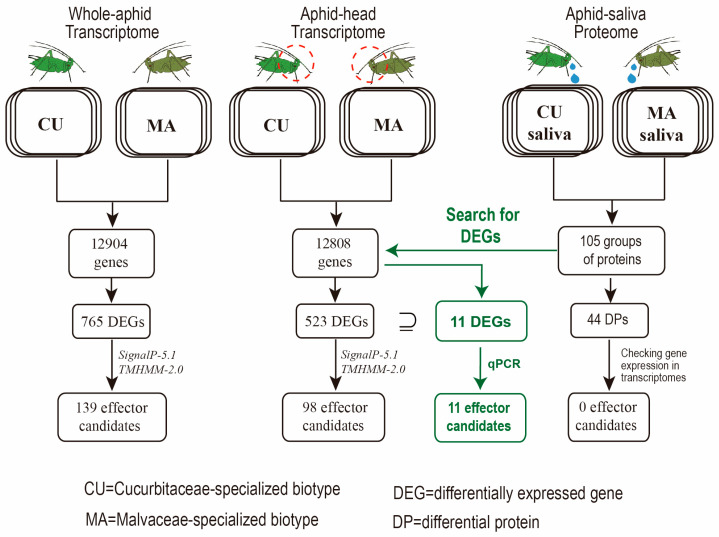
The strategies used to identify biotype-specific effector candidates of *A. gossypii* in this study. The colored pipelines showed the most efficient strategy.

In this study, we tried three strategies to identify biotype-specific salivary effectors that were hypothesized to determine the host specialization of *A. gossypii*. The comparative transcriptome analysis of whole aphids of CU and MA biotypes identified 139 effector candidates, and the analysis of aphid heads identified 98 biotype-specific effector candidates. To further refine the catalog of effector candidates, we conducted a comparative quantitative proteomic analysis of aphid saliva. A total of 105 groups of proteins (representing 177 possible proteins) were identified, 44 of which were different in abundance between the CU and MA biotype saliva collected from the artificial diet. Unfortunately, none of the 44 proteins were significantly differently expressed between the two biotypes. Finally, we used a strategy in which the salivary proteins with transcriptional differences between the two biotypes were considered as the final biotype-specific effectors. Through this strategy, 11 biotype-specific effectors were obtained, and their gene expression differences were all confirmed by RT-qPCR. Compared with other strategies, the combined analysis had the highest efficiency in identifying biotype-specific effector candidates (Figure 3).

Aphid effectors were mainly expressed in aphid salivary glands [20,21] and were secreted into saliva and delivered into plant tissues. In addition, aphid effectors are most likely classically secreted proteins [53,54]. Based on that knowledge, transcriptomic approaches were widely used to identify effector candidates in aphids and other sap-sucking insects. For instance, the analysis of the transcriptomic data of *A. pisum* salivary glands (SG) identified 12,040 genes encoding proteins, among which 3603 had the features of secreted proteins and were regarded as SG-expressed effector candidates [25]. Comparative transcriptome analysis of head and alimentary tract tissues identified 1989 SG up-regulated genes, among which 740 genes encoding secreted proteins [25]. Similarly, through comparative transcriptome analysis of aphid heads and other tissues, 725 genes encoding putatively secreted proteins were identified and were up-regulated in head tissues of the Russian wheat aphid *Diuraphis noxia* [34]. In the present study, we used comparative transcriptome analysis to identify biotype-specific effector candidates in *A. gossypii*. Different from previous studies, we conducted a comparative analysis between two biotypes of *A. gossypii*. The comparative analysis of whole-aphid transcriptomes identified 139 biotype-specific effector candidates, and that of aphid-head transcriptomes identified 98 biotype-specific effector candidates, which is consistent with the fact that effectors or elicitors are mainly expressed in the salivary glands located in aphid head. Those biotype-specific effector candidates from *A. gossypii* were much less than common effector candidates identified from other aphid species [25,34]. Theoretically, comparative analysis of dissected salivary glands should generate a smaller repertoire of putative effector proteins. However, dissection of aphid salivary glands is not only difficult but also may interfere with the quality of RNA-seq because some mRNA would degrade during dissection. 

Homology analysis based on published aphid effectors is an alternative strategy to predict saliva effectors. For example, C002 was detected in *A. pisum*-exposed plants [17], and the expression of *M. persicae* C002 in plants promoted *M. persicae* reproduction [20]. Silence of the homologous gene of C002 in *Schizaphis graminum* led to the lethality of the aphid on wheat plants [54]. In this study, we found homologous proteins of 15 published aphid effectors in *A. gossypii*. However, none of them were differentially expressed between CU and MA biotypes. It was uncertain whether those homologous proteins could promote the infestation of *A. gossypii* on their native hosts, but at least we could conclude that they did not determine the host-specialization of *A. gossypii* because they were not expressed differentially between the two biotypes. There was a study demonstrating that the genes specifically expressed in aphid salivary glands experienced faster evolutions than other genes [33]. For this reason, we do not think aphid salivary effectors are likely obtained through homologous analysis of published effectors.

Transcriptome analysis identified nearly 100 effector candidates, but functional characterization of those effectors still remains a daunting task. Quantitative proteome provided an efficient choice for identifying effector candidates directly from aphid saliva [55]. A range of effector candidates was identified from a number of aphids, such as *M. euphorbiae* [21], *A. pisum* [25], and *Schizaphis graminum* [56]. We identified 105 protein groups that represented 177 possible proteins in *A. gossypii* saliva. Comparisons with the salivary proteomic data of 10 other aphid species indicated that 38 of the 105 protein groups had been identified at least in one of the 10 aphid species. We obtained 44 differential proteins between the CU and MA biotypes. Of the 44 proteins, 10 proteins were unique to the CU biotype, and 8 were unique to the MA biotype. The proteomics approach was more efficient than the transcriptomics approach in identifying biotype-specific effectors. However, out of our expectation, none of the genes of the 44 differential proteins were differentially expressed between the two biotypes. One of the reasons may be that the composition of salivary proteins collected from the artificial diet was distorted from that injected into plants during aphid feeding. Studies reported that a significant change in salivary secretions was observed when aphids fed on different artificial diets or in different feeding stages [10,49]. In addition, some salivary proteins that were injected into plant tissues when aphids fed on plants might be rarely secreted into an artificial diet [36]. Therefore, it may be problematic to identify biotype-specific effectors by the quantitative comparison of salivary proteins identified from saliva from different aphid biotypes. In order to make full use of the salivary proteome data, we searched for DEGs of those salivary proteins in the aphid-head transcriptomes. The salivary proteins with differential gene expressions between the two biotypes were regarded as biotype-specific effector candidates. By this strategy, we obtained as few as a dozen biotype-specific effector candidates. This method can avoid the problem of possible distortion of the composition of aphid saliva collected in an artificial diet. More advantageous, saliva collected from different biotypes of aphids can be pooled for a single mass spectrographic analysis, which can greatly reduce the cost. However, for this strategy, it is necessary to collect as much saliva as possible to ensure that all protein components are included in the artificial diet. In addition, this strategy may have some flaws because there are opportunities for regulation after transcription that could lead to differential protein levels independent of mRNA levels [57]. In other words, differential transcriptions may not lead to differential proteins, or differential proteins may not derive from differential transcriptions. Therefore, salivary proteins that were excluded from the effector candidates by this strategy may also be worth considering.

The contamination of non-aphid proteins in aphid saliva may interfere with the result of salivary proteomic analysis. We collected aphid saliva using the same method as described previously [42,58,59] by collecting the artificial diet on which aphids had been fed for 24 h. During the feeding process, aphid stylet introduced some bacteria into the artificial diet (Wang et al., unpublished data). Although the artificial diet containing salivary proteins was filtered to remove bacterial cells prior to proteomic analysis, the filter could not get rid of proteins produced by those bacteria within 24 h of feeding. Thus, the aphid saliva we collected probably was a mix of aphid and non-aphid proteins. Therefore, the 105 protein groups (representing 177 possible proteins) identified in this study might include proteins from bacteria. In order to reduce the possible contamination, we suggested shortening the saliva sampling time to 12 h or less to reduce possible contaminant proteins produced by oral bacteria of aphids. 

In the present study, we only emphasized proteins that were differentially expressed between host-specialized biotypes. Effectors were defined as molecules secreted by plant-associated organisms, such as bacteria, fungi, oomycetes, nematodes, and aphids, that alter plant-cell structure and functional process [60]. Effectors were proteins and/or small molecules that were secreted due to the interaction with the host cells [61]. Therefore, we probably overlooked small non-protein molecules in the aphid saliva, such as peptides [62], RNAs [63], and even hormones [64]. These non-protein small molecules may also play a key role in the host-specialization of aphids. In addition, an aphid was, in fact, a symbiosis of the aphid and endosymbionts, and the endosymbionts represented 2–5% of the total biomass of the symbiosis [65]. There were endosymbiont-originated proteins in aphid saliva, and some of those proteins, such as GroEL, played an important role in the aphid–plant interactions [65]. Symbiotic bacteria of aphids of different biotypes may express different proteins, which causes aphid host-specialization. Therefore, in the future, attention needs to be paid to those bacteria-originated proteins and tests performed regarding their functions in the host-specialization of aphids.

In this research, we tried three strategies to identify putative biotype-specific effector proteins. The transcriptomic approaches have the advantage of high reliability, and the proteomic approaches have the advantage of high efficiency. The optimal strategy we established here combined the two advantages and identified as few as a dozen effector candidates. This strategy avoids the tedious task of dissecting aphid salivary glands. This strategy can be used to identify differential salivary proteins not only between host-specialized aphid biotypes but also between virulent ecotypes of other sap-sucking insects.

## Figures and Tables

**Table 2:** Comparison of salivary proteins identified in *A. gossypii* with those identified in 10 other aphid species.

Salivary Protein Identified in *A. gossypii*	Aphid Species									
	*Sa*	*Ap*	*Mp*	*Mc*	*Rp*	*Sg*	*Me*	*Sc*	*Ac*	*Dn*
Actin	✓	✓	✓	✓	✓		✓	✓	✓	
Glucose dehydrogenase	✓	✓	✓	✓	✓	✓	✓		✓	✓
Peroxidase	✓	✓	✓	✓	✓		✓			
Heat shock protein	✓		✓				✓		✓	
Carboxypeptidase					✓					
14-3-3 protein			✓				✓			
EF1-α	✓	✓	✓						✓	
40S ribosomal protein	✓									
60S ribosomal protein	✓									
Endochitinase									✓	
Calreticulin										✓
Histone	✓	✓	✓				✓	✓	✓	
Carbonic anhydrase	✓		✓	✓	✓	✓	✓		✓	
Apolipophorins	✓	✓	✓		✓		✓			
ATP synthase	✓		✓				✓		✓	
Tubulin	✓		✓					✓	✓	
ASC1	✓									
MINPP					✓					
RNA-binding protein						✓				
LOC114119699	✓						✓			
LOC114120354	✓	✓	✓		✓		✓			
LOC114128856	✓						✓			
LOC114129844						✓	✓			
LOC114124500	✓		✓	✓	✓		✓			
PF11_0213-like							✓			
LOC114124821							✓			
LOC114118907		✓	✓	✓	✓					
LOC114119100					✓					
LOC114119139							✓			
LOC114124575	✓	✓	✓				✓			
LOC114133079	✓	✓	✓	✓	✓	✓	✓		✓	✓
LOC114123730	✓	✓	✓		✓		✓			
LOC114123729	✓	✓	✓				✓			
LOC114128009	✓	✓	✓				✓		✓	
LOC114123311					✓		✓			
LOC114121223		✓					✓			
LOC114130459	✓	✓					✓			
DDB_G0267840					✓					

‘✓’ indicates identified proteins. MINPP, multiple inositol polyphosphate phosphatase; ASC1, activating signal cointegrator 1. *Sa*, *Sitobion avenae* [49]; *Ap*, *Acyrthosiphon pisum* [25]; *Mp*, *Myzus persicae* [32]; *Mc*, *Myzus cerasi* [32]; *Rp*, *Rhopalosiphum padi* [32]; *Sg*, *Schizaphis graminum* [50]; *Me*, *Macrosiphum euphorbiae* [36]; *Sc*, *Schlectendalia chinensis* [41]; *Ac*, *Aphis craccivora* [51]; *Dn*, *Diuraphis noxia* [52].

**Table 3:** Salivary proteins differentially expressed between CU and MA biotypes of *A. gossypii*.

Protein ID	Gene ID	Description	Regulate ^#^	*FC* ^¶^	Signal Peptide
XP_027839590.1	LOC114121443	actin	down	0.43	no
XP_027854514.1	LOC114133090	heat shock protein	down	0.38	no
XP_027853233.1	LOC114132056	heat shock protein	down	0.46	no
XP_027840464.1	LOC114122099	heat shock protein	down	0.49	no
XP_027849246.1	LOC114128852	histone H3.3	up	1.31	no
XP_027845398.1	LOC114125817	apoptosis-stimulating protein	up	1.24	no
XP_027850174.1	LOC114129582	uncharacterized	down	0.67	yes
XP_027851813.1	LOC114130922	E3 ubiquitin-protein ligase	up	1.27	no
XP_027837426.1	LOC114119891	uncharacterized	up	2.42	yes
XP_027836138.1	LOC114118907	uncharacterized	down	3.43	yes
XP_027852041.1	LOC114131096	uncharacterized	down	0.12	yes

^#^ Comparison of CU biotype with MA biotype; ^¶^ *FC*, the fold of change.

## Data Availability

Raw sequencing data are available in NCBI Sequence Read Archive with reference number PRJNA899574.

## Supplementary Materials

The following supporting information can be downloaded at https://www.mdpi.com/article/10.3390/insects14090760/s1, Figure S1: Phylogenetic analysis of effector candidates XP027837426.1; Table S1: Primers used in RT-qPCR for validation of DEGs; Table S2: Biotype-specific effector candidates from whole-aphid transcriptome; Table S3: Biotype-specific effector candidates from aphid-head transcriptome; Table S4: Protein groups identified from aphid saliva by mass Spectrometry; Table S5: Biotype-specific protein groups identified by label-free quantification.

## References

[1] Blackman R.L., Eastop V.F. (2000). Aphids on the World’s Crops: An Identification and Information Guide.

[2] Peccoud J., Simon J.-C., von Dohlen C., D’acier A.C., Plantegenest M., Vanlerberghe-Masutti F., Jousselin E. (2010). Evolutionary history of aphid-plant associations and their role in aphid diversification. C. R. Biol..

[3] Guldemond J.A., Tigges W.T., De Vrijer P.W.F. (1994). Host Races of Aphis gossypii (*Homoptera: Aphididae*) on Cucumber and Chrysanthemum. Environ. Èntomol..

[4] Liu X.-D., Xu T.-T., Lei H.-X. (2017). Refuges and host shift pathways of host-specialized aphids Aphis gossypii. Sci. Rep..

[5] Carletto J., Lombaert E., Chavigny P., Brévault T., Lapchin L., Vanlerberghe-Masutti F. (2009). Ecological specialization of the aphid *Aphis gossypii* Glover on cultivated host plants. Mol. Ecol..

[6] Wang L., Zhang S., Luo J.-Y., Wang C.-Y., Lv L.-M., Zhu X.-Z., Li C.-H., Cui J.-J. (2016). Identification of *Aphis gossypii* Glover (Hemiptera: Aphididae) Biotypes from Different Host Plants in North China. PLoS ONE.

[7] Will T., Kornemann S.R., Furch A.C., Tjallingii W.F., van Bel A.J. (2009). Aphid watery saliva counteracts sieve-tube occlusion: A universal phenomenon?. J. Exp. Biol..

[8] Will T., Furch A.C.U., Zimmermann M.R. (2013). How phloem-feeding insects face the challenge of phloem-located defenses. Front. Plant Sci..

[9] Vandermoten S., Harmel N., Mazzucchelli G., De Pauw E., Haubruge E., Francis F. (2014). Comparative analyses of salivary proteins from three aphid species. Insect Mol. Biol..

[10] Zhang Y., Liu X., Fu Y., Crespo-Herrera L., Liu H., Wang Q., Zhang Y., Chen J. (2022). Salivary Effector Sm9723 of Grain Aphid *Sitobion miscanthi* Suppresses Plant Defense and Is Essential for Aphid Survival on Wheat. Int. J. Mol. Sci..

[11] Kuśnierczyk A., Winge P., Jørstad T.S., Troczyńska J., Rossiter J.T., Bones A.M. (2008). Towards global understanding of plant defence against aphids--timing and dynamics of early Arabidopsis defence responses to cabbage aphid (*Brevicoryne brassicae*) attack. Plant Cell Environ..

[12] Louis J., Shah J. (2015). Plant defence against aphids: The PAD4 signalling nexus. J. Exp. Bot..

[13] Moran P.J., Cheng Y., Cassell J.L., Thompson G.A. (2002). Gene expression profiling of Arabidopsis thaliana in compatible plant-aphid interactions. Arch. Insect Biochem. Physiol..

[14] Hogenhout S., Bos J.I. (2011). Effector proteins that modulate plant–insect interactions. Curr. Opin. Plant Biol..

[15] Snoeck S., Guayazán-Palacios N., Steinbrenner A.D. (2022). Molecular tug-of-war: Plant immune recognition of herbivory. Plant Cell.

[16] Karban R. (2019). The ecology and evolution of induced responses to herbivory and how plants perceive risk. Ecol. Èntomol..

[17] Mutti N.S., Louis J., Pappan L.K., Pappan K., Begum K., Chen M.-S., Park Y., Dittmer N., Marshall J., Reese J.C. (2008). A protein from the salivary glands of the pea aphid, *Acyrthosiphon pisum*, is essential in feeding on a host plant. Proc. Natl. Acad. Sci. USA.

[18] Cui N., Lu H., Wang T., Zhang W., Kang L., Cui F. (2019). Armet, an aphid effector protein, induces pathogen resistance in plants by promoting the accumulation of salicylic acid. Philos. Trans. R. Soc. B Biol. Sci..

[19] Wang Z., Lü Q., Zhang L., Zhang M., Chen L., Zou S., Zhang C., Dong H. (2021). Aphid salivary protein Mp1 facilitates infestation by binding phloem protein 2-A1 in Arabidopsis. Biochem. Biophys. Res. Commun..

[20] Bos J.I.B., Prince D., Pitino M., Maffei M.E., Win J., Hogenhout S.A. (2010). A Functional Genomics Approach Identifies Candidate Effectors from the Aphid Species Myzus persicae (Green Peach Aphid). PLoS Genet..

[21] Atamian H.S., Chaudhary R., Cin V.D., Bao E., Girke T., Kaloshian I., Luna E., van Eck L., Campillo T., Weinroth M. (2013). In Planta Expression or Delivery of Potato Aphid *Macrosiphum euphorbiae* Effectors *Me10* and *Me23* Enhances Aphid Fecundity. Mol Plant Microbe Interact.

[22] Rodriguez P.A., Escudero-Martinez C., Bos J.I. (2017). An Aphid Effector Targets Trafficking Protein VPS52 in a Host-Specific Manner to Promote Virulence. Plant Physiol..

[23] Kanvil S., Powell G., Turnbull C. (2014). Pea aphid biotype performance on diverse Medicago host genotypes indicates highly specific virulence and resistance functions. Bull. Entomol. Res..

[24] Pitino M., Hogenhout S.A. (2013). Aphid protein effectors promote aphid colonization in a plant species-specific manner. Mol. Plant Microbe Interact..

[25] Boulain H., Legeai F., Jaquiéry J., Guy E., Morlière S., Simon J.-C., Sugio A. (2019). Differential Expression of Candidate Salivary Effector Genes in Pea Aphid Biotypes With Distinct Host Plant Specificity. Front. Plant Sci..

[26] Liu X.D., Zhai B., Zhang X. (2002). Studies on the host biotypes and its cause of cotton aphid in Nanjing, China. Agric. Sci. China.

[27] Liu X.D., Zhai B., Zzhang X., Yang L. (2004). Differentiation in morphometrics and ecological adaptability of cotton and cucumber biotypes of the cotton aphid, *Aphis gossypii* (Homoptera: Aphididae). Acta Entomol. Sin..

[28] Najar-Rodríguez A.J., McGRAW E.A., Hull C.D., Mensah R.K., Walter G.H. (2009). The ecological differentiation of asexual lineages of cotton aphids: Alate behaviour, sensory physiology, and differential host associations. Biol. J. Linn. Soc..

[29] Ali F., Hu X., Wang D., Yang F., Guo H., Wang Y. (2021). Plant pathogen-mediated rapid acclimation of a host-specialized aphid to a non-host plant. Ecol. Evol..

[30] Quan Q., Hu X., Pan B., Zeng B., Wu N., Fang G., Cao Y., Chen X., Li X., Huang Y. (2018). Draft genome of the cotton aphid Aphis gossypii. Insect Biochem. Mol. Biol..

[31] Zhang S., Gao X., Wang L., Jiang W., Su H., Jing T., Cui J., Zhang L., Yang Y. (2022). Chromosome-level genome assemblies of two cotton-melon aphid *Aphis gossypii* biotypes unveil mechanisms of host adaption. Mol. Ecol. Resour..

[32] Thorpe P., Cock P.J.A., Bos J. (2016). Comparative transcriptomics and proteomics of three different aphid species identifies core and diverse effector sets. BMC Genom..

[33] Boulain H., Legeai F., Guy E., Morlière S., Douglas N.E., Oh J., Murugan M., Smith M., Jaquiéry J., Peccoud J. (2018). Fast Evolution and Lineage-Specific Gene Family Expansions of *Aphid Salivary* Effectors Driven by Interactions with Host-Plants. Genome. Biol. Evol..

[34] Nicolis V.F., Burger N.F.V., Botha A.-M. (2022). Whole-body transcriptome mining for candidate effectors from *Diuraphis noxia*. BMC Genom..

[35] Carolan J.C., Fitzroy C.I.J., Ashton P.D., Douglas A.E., Wilkinson T.L. (2009). The secreted salivary proteome of the pea aphid Acyrthosiphon pisum characterised by mass spectrometry. PROTEOMICS.

[36] Chaudhary R., Atamian H.S., Shen Z., Briggs S.P., Kaloshian I. (2015). Potato Aphid Salivary Proteome: Enhanced Salivation Using Resorcinol and Identification of Aphid Phosphoproteins. J. Proteome Res..

[37] Dobin A., Davis C.A., Schlesinger F., Drenkow J., Zaleski C., Jha S., Batut P., Chaisson M., Gingeras T.R. (2013). STAR: Ultrafast universal RNA-seq aligner. Bioinformatics.

[38] Liao Y., Smyth G.K., Shi W. (2013). feature Counts: An efficient general purpose program for assigning sequence reads to genomic features. Bioinformatics.

[39] Nielsen H. (2017). Predicting Secretory Proteins with SignalP. Methods Mol Biol..

[40] Krogh A., Larsson B., von Heijne G., Sonnhammer E.L. (2001). Predicting transmembrane protein topology with a hidden markov model: Application to complete genomes. J. Mol. Biol..

[41] Yang Z., Ma L., Francis F., Yang Y., Chen H., Wu H., Chen X. (2018). Proteins Identified from Saliva and Salivary Glands of the Chinese Gall Aphid *Schlechtendalia chinensis*. PROTEOMICS.

[42] Cooper W.R., Dillwith J.W., Puterka G.J. (2010). Salivary Proteins of Russian Wheat Aphid (Hemiptera: Aphididae). Environ. Èntomol..

[43] van Bel A.J., Will T. (2016). Functional Evaluation of Proteins in Watery and Gel Saliva of Aphids. Front. Plant Sci..

[44] Wang W., Dai H., Zhang Y., Chandrasekar R., Luo L., Hiromasa Y., Sheng C., Peng G., Chen S., Tomich J.M. (2015). Armet is an effector protein mediating aphid-plant interactions. FASEB J..

[45] Kettles G.J., Kaloshian I. (2016). The Potato Aphid Salivary Effector Me47 Is a Glutathione-S-Transferase Involved in Modifying Plant Responses to Aphid Infestation. Front. Plant Sci..

[46] MacWilliams J.R., Dingwall S., Chesnais Q., Sugio A., Kaloshian I. (2020). AcDCXR Is a Cowpea Aphid Effector With Putative Roles in Altering Host Immunity and Physiology. Front. Plant Sci..

[47] Rodriguez P.A., Stam R., Warbroek T., Bos J.I.B. (2014). Mp10 and Mp42 from the Aphid Species *Myzus persicae* Trigger Plant Defenses in *Nicotiana benthamiana* Through Different Activities. Mol. Plant Microbe Interact..

[48] Wang W., Luo L., Lu H., Chen S., Kang L., Cui F. (2015). Angiotensin-converting enzymes modulate aphid–plant interactions. Sci. Rep..

[49] Zhang Y., Fu Y., Francis F., Liu X., Chen J. (2021). Insight into watery saliva proteomes of the grain aphid, *Sitobion avenae*. Arch. Insect Biochem. Physiol..

[50] Nicholson S.J., Puterka G.J. (2014). Variation in the salivary proteomes of differentially virulent greenbug (Schizaphis graminum Rondani) biotypes. J. Proteom..

[51] Loudit S.M.B., Bauwens J., Francis F. (2018). Cowpea aphid-plant interactions: Endosymbionts and related salivary protein patterns. Èntomol. Exp. Appl..

[52] Nicholson S.J., Hartson S.D., Puterka G.J. (2012). Proteomic analysis of secreted saliva from Russian Wheat Aphid (Diuraphis noxia Kurd.) biotypes that differ in virulence to wheat. J. Proteom..

[53] Kaloshian I., Walling L.L. (2015). Hemipteran and dipteran pests: Effectors and plant host immune regulators. J. Integr. Plant Biol..

[54] Zhang Y., Fan J., Sun J.-R., Chen J.-L. (2015). Cloning and RNA interference analysis of the salivary protein C002 gene in Schizaphis graminum. J. Integr. Agric..

[55] Carolan J.C., Caragea D., Reardon K.T., Mutti N.S., Dittmer N., Pappan K., Cui F., Castaneto M., Poulain J., Dossat C. (2011). Faculty Opinions recommendation of Predicted effector molecules in the salivary secretome of the pea aphid (Acyrthosiphon pisum): A dual transcriptomic/proteomic approach. J. Proteome Res..

[56] Zhang Y., Liu X., Francis F., Xie H., Fan J., Wang Q., Liu H., Sun Y., Chen J. (2022). The salivary effector protein Sg2204 in the greenbug *Schizaphis graminum* suppresses wheat defence and is essential for enabling aphid feeding on host plants. Plant Biotechnol. J..

[57] Will T., Tjallingii W.F., Thönnessen A., van Bel A.J.E. (2007). Molecular sabotage of plant defense by aphid saliva. Proc. Natl. Acad. Sci. USA.

[58] DE Vos M., Jander G. (2009). *Myzus persicae* (green peach aphid) salivary components induce defence responses in *Arabidopsis thaliana*. Plant Cell Environ..

[59] Hogenhout S.A., Van der Hoorn R.A.L., Terauchi R., Kamoun S. (2009). Emerging Concepts in Effector Biology of Plant-Associated Organisms. Mol. Plant Microbe Interact..

[60] Mondal H.A. (2020). Aphid saliva: A powerful recipe for modulating host resistance towards aphid clonal propagation. Arthropod-Plant Interact..

[61] Londono-Renteria B., Drame P.M., Montiel J., Vasquez A.M., Tobón-Castaño A., Taylor M., Vizcaino L., Lenhart A.E. (2020). Identification and Pilot Evaluation of Salivary Peptides from *Anopheles albimanus* as Biomarkers for Bite Exposure and Malaria Infection in Colombia. Int. J. Mol. Sci..

[62] Chen Y., Singh A., Kaithakottil G.G., Mathers T.C., Gravino M., Mugford S.T., van Oosterhout C., Swarbreck D., Hogenhout S.A. (2020). An aphid RNA transcript migrates systemically within plants and is a virulence factor. Proc. Natl. Acad. Sci. USA.

[63] Acevedo F.E., Smith P., Peiffer M., Helms A., Tooker J., Felton G.W. (2019). Phytohormones in Fall Armyworm Saliva Modulate Defense Responses in Plants. J. Chem. Ecol..

[64] Whitehead L.F., Douglas A.E. (1993). Populations of symbiotic bacteria in the parthenogenetic pea aphid (*Acyrthosiphon pisum*) symbiosis. Proc. R. Soc. B Boil. Sci..

[65] Chaudhary R., Atamian H.S., Shen Z., Briggs S.P., Kaloshian I. (2014). GroEL from the endosymbiont *Buchnera aphidicola* betrays the aphid by triggering plant defense. Proc. Natl. Acad. Sci. USA.

