# Supplementary material for: A Method for Identification of Biotype-Specific Salivary Effector Candidates of Aphid
Source: Insects. 2023 Sep 13;14(9):760. doi: 10.3390/insects14090760 (PMC10532216; doi:10.3390/insects14090760)
Supplement: Supplementary file 1 [file insects-14-00760-s001.zip › Figure S1 Phylogenetic analysis of effector candidates XP027837426.1.pdf]

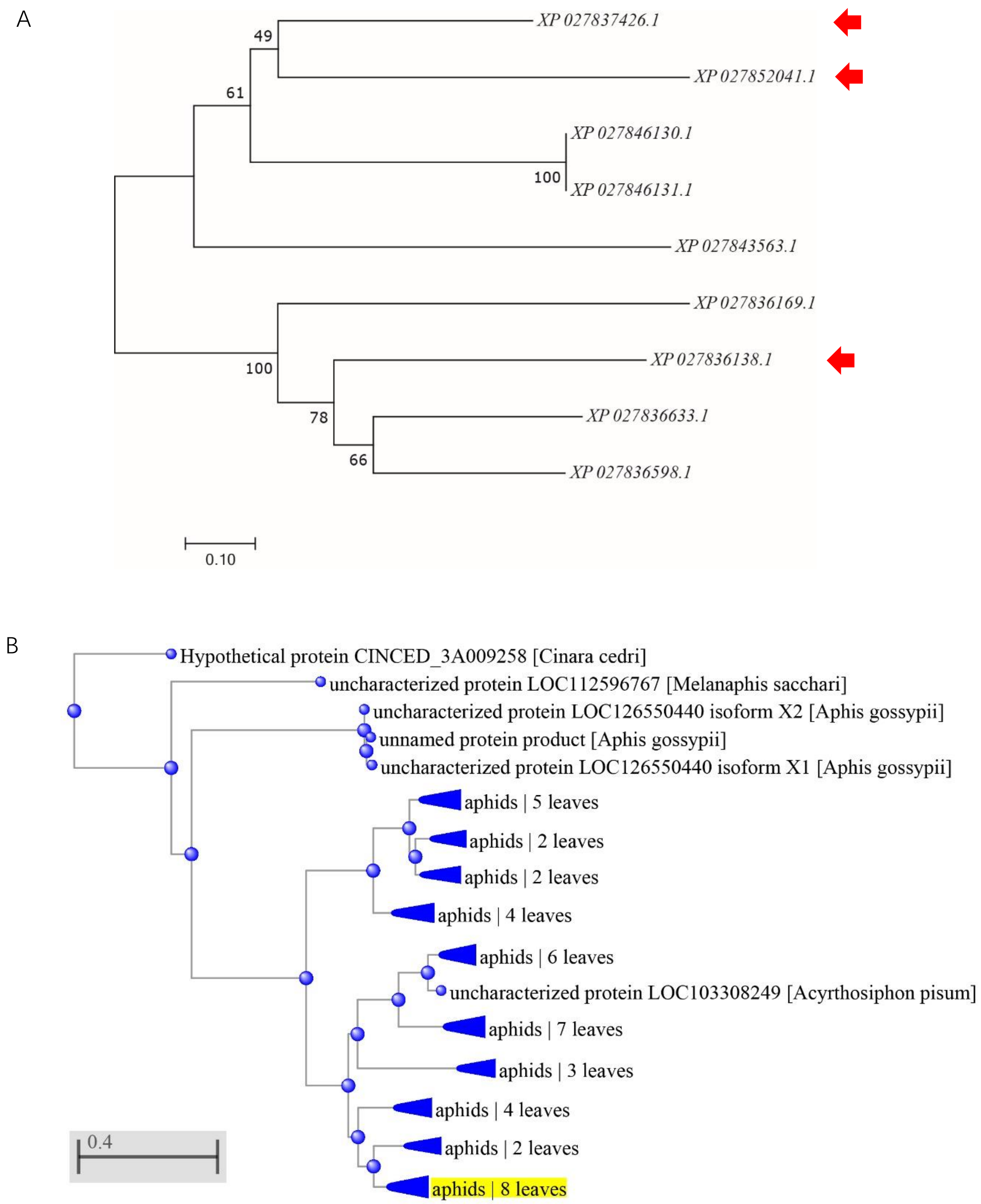

Figure S1. Phylogenetic analysis of effector candidates XP027837426.1. A, Neighbor joining tree of BLASTP hits of XP027837426.1 in *Aphis gossypii*, the red arrow indicates effector candidates identified in the study; B, Fast minimum evolution tree of the first 50 hits of XP027837426.1, XP027837426.1 was included in the yellow branch.
