# Supplementary material for: A Method for Identification of Biotype-Specific Salivary Effector Candidates of Aphid
Source: Insects. 2023 Sep 13;14(9):760. doi: 10.3390/insects14090760 (PMC10532216; doi:10.3390/insects14090760)
Supplement: Supplementary file 1 [file insects-14-00760-s001.zip › Table S1 Primers used in RT-qPCR for validation of DEGs.pdf]

Table S1. Primers used in RT-qPCR for validation of differentially expressed genes (DEGs).

Gene ID	Forward primer (5'–3')	Reverse primer (5'–3')
LOC114121443	TAAAACCGTCGGTACCACCC	ACAACCAAAGGCGCAACATC
LOC114133090	CAAAGTGGTGAACGACAGCG	GTCACTTCACGGCCCAAGTA
LOC114132056	CAGTGCGACCGAAACACATTA	CGCAATAACGCACAATTCGATG
LOC114122099	GGAACGCGGGTAAACAGGAA	CGAGAACCGGGGAAACCAC
LOC114128852	TGCAGTTCATTGCCGTGTTG	AGCCATGGTTCAAGAGTACGA
LOC114125817	TTATCGACGACCAACTGCCC	CAGTTCTGTGCCTGTTTCGC
LOC114129582	AAACCATTTTCCTGCTCGGG	GAGGTGCTGGTGGTACTCTA
LOC114130922	TCGCGGTGATTAGGTGATCG	TCGACGACGAGGCGTTAAAA
LOC114119891	CGTGGTGTGCTGACAATCAA	GCTACGAATCCGCGTGCATA
LOC114118907	AGGGGCAATTTTTCCGTGAAC	CGTCAAACTTCGCACATGGT
LOC114131096	CGCCCATGCACCAGATTTTG	TCTCGCAGTCGGTGATAGGA

Ef1-α	TATGGTGGTTCAGTAGAGTC	CTGATTGTGCCGTGCTTATTG
